# Automated Nanocrystalline Sponge Workflow Enabled by 3D Electron Diffraction

**DOI:** 10.1021/jacs.5c21773

**Published:** 2026-03-05

**Authors:** Sofiia Butonova, Yinlin Chen, Jung Cho, Marcus Wallin, Zhehao Huang, Xiaodong Zou

**Affiliations:** Department of Chemistry, 7675 Stockholm University, Stockholm SE-106 91, Sweden

## Abstract

The crystalline sponge (CS) method utilizes a crystalline porous material to arrange target molecules within its periodic pores. This enables the determination of the 3D atomic structures of organic molecules without the need for crystallization. However, its applicability is currently limited by the availability of suitable porous single crystals that can grow to a sufficient size for X-ray diffraction analysis. Although three-dimensional electron diffraction (3D ED) allows structure determination from nanosized crystals, ab initio structural analysis of organic molecules hosted in nanocrystalline sponges remains challenging and largely manual. Here, we present a 3D ED-based nanocrystalline sponge (NanoCS) workflow that integrates guest soaking, low-dose cryogenic data collection, and automated structure solution and refinement. A key advance is a newly developed automated approach for guest identification and structural analysis implemented in the AutoSolveX pipeline. Using the nanocrystalline bismuth-based metal–organic framework (MOF) SU-100 as a prototype crystalline sponge, we demonstrated the general applicability of this NanoCS strategy. 10 organic molecules, introduced as pure liquids, solutions, or vapors, are investigated. For all systems, 3D ED data collected under low electron fluence and cryogenic conditions enabled fully automated identification and refinement of the guest molecules using AutoSolveX. The results confirm the periodic arrangement of the guest molecules within the pores of SU-100, mediated by coordination bonding, hydrogen bonding, offset π–π stacking, and van der Waals interactions. This work establishes NanoCS combined with automated structural analysis as a practical and high-throughput platform for routine ab initio structural determination of organic molecules from nanocrystalline hosts.

## Graphical abstract

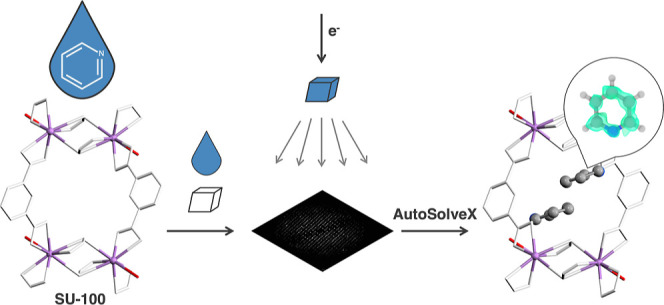

## Introduction

The three-dimensional (3D) structures of most organic molecules are determined by X-ray diffraction. However, this method requires large single crystals, which are often challenging or impossible to grow. In 2013, Fujita’s group introduced the crystalline sponge (CS) method, which enables the structure determination of organic molecules using crystallographic techniques without the need to crystallize the target compounds.^1–9^ In this approach, target molecules are adsorbed and aligned within the ordered pores of a crystalline porous host that serves as the sponge, achieving the long-range ordering required for single-crystal X-ray diffraction (SCXRD). The CS method is particularly valuable for analyzing molecules that are difficult to crystallize such as oily and volatile compounds. It can also determine the absolute configuration of chiral molecules.^10^ Since only a few host crystals are needed, the CS method is well-suited for analyzing trace samples, including natural products available in minute quantities.^11^

In principle, any crystalline porous material that can host and order guest molecules is a potential CS candidate.^1,12–20^ Among such hosts, metal–organic frameworks (MOFs) are particularly promising, attributed to their structural diversity, tunable pore geometries, and chemically functionalizable linkers that can be tailored to optimize host–guest interactions. The first reported crystalline sponge, introduced by Fujita et al. in 2013, was a Zn-based MOF [(ZnI_2_)_3_(tpt)_2_·*x*(Guest)]_*n*_ [tpt = 2,4,6-tris­(4-pyridyl)-1,3,5-triazine]. This MOF crystallizes in a monoclinic structure with pore dimensions of 5 Å × 8 Å and has a flexible framework that can accommodate a wide range of molecules with different sizes and varying functionalities.^11^ However, this Zn-MOF is desolvation-sensitive, which complicates the complete removal of solvent molecules (e.g., cyclohexane). Residual solvents may hinder guest uptake or contribute to the disorder of guest molecules in the pores. In 2016, Yaghi’s group reported a chiral Al-based MOF, MOF-520, as a crystalline sponge and introduced the coordinative alignment (CAL) method, in which guest molecules align within the pores through coordination bonds.^2^ This approach enabled structural determination of various molecules, including assignment of their absolute configurations.^2,21^

These studies highlight the power of the CS method for the ab initio determination of 3D atomic structures, regardless of the physical state of the target molecules. However, a key drawback of the current CS method using SCXRD is the requirement of large (>50 μm), high-quality single crystals. These large crystals are prone to cracking during solvent exchange and guest soaking, compromising SCXRD data quality. As a result, guest-soaking is considered the most critical step, demanding substantial effort to optimize guest-soaking conditions.^9^ Slow guest uptake, often requiring several days, is usually necessary to minimize crystal damage.^21^ Consequently, the CS method has seen limited widespread adoption, and only a handful of MOFs have been identified as suitable crystalline sponges.^1,12–18^

Three-dimensional electron diffraction (3D ED) has emerged as a powerful technique for structural determination of nanocrystalline materials, including MOFs, which often crystallize as submicrometer-sized crystals unsuited for SCXRD.^22–25^ By overcoming the size limitations of SCXRD, 3D ED could extend the applicability of the CS method. Moreover, using smaller crystals improves guest diffusion and reduces the risk of cracking during soaking and handling. Additionally, 3D ED enables rapid crystal screening on a transmission electron microscope (TEM) that facilitates the selection of high-quality crystals for data collection. These advantages suggest that a nanocrystalline sponge (nanoCS) approach, integrating nanosized crystals with 3D ED, could overcome current limitations and significantly expand the CS method.

Despite these advantages, the structure determination of guest organic molecules within porous materials by 3D ED remains challenging. While structural analyses of organic solvents in MOF CAU-36 ^26^ and organic structural directing agents in the open-framework germanates^27^ by 3D ED were reported, only two examples have demonstrated structure determination of guest molecules through MOF crystalline sponges, [(ZnI_2_)_3_(tpt)_2_][Bibr ref28] and SIMOF-5.^29^ The host–guest interactions are via coordination bonding for SIMOF-5 ^29^ and hydrogen bonding for [(ZnI_2_)_3_(tpt)_2_].^28^ Electron irradiation can damage both host and guest species through ionization and bond cleavage, potentially disrupting ordered host–guest interactions. Furthermore, the high-vacuum environment of TEM may induce guest desorption, complicating the reliable structure determination. Even when suitable data are obtained, guest identification and refinement are typically manual and labor-intensive, limiting throughput and reproducibility. To address these challenges, both experimental and analytical advances are required. 3D ED data collection conditions must be optimized to preserve guest molecules in their adsorbed state, and systematic studies are needed to understand how guests’ physical state, functional groups, and soaking conditions influence incorporation and ordering. Equally important is the development of high-throughput workflows that automate guest identification and structure refinement from 3D ED data, particularly for studies involving multiple guest molecules.

Here, we present a 3D ED-based nanocrystalline sponge (NanoCS) pipeline that integrates guest soaking, low-dose cryogenic data collection, and automated structure determination (Figure 1). We have developed a new automated approach for guest identification and structure determination, implemented in an integrated structure-solution and refinement workflow. Using a flexible bismuth-based MOF, SU-100, as a prototype crystalline sponge, we demonstrate the feasibility of NanoCS and systematically evaluate key experimental and structural parameters governing successful implementation. Together, these results establish NanoCS as a high-throughput platform for structure determination of organic molecules from nanocrystalline hosts.

**Figure 1:**
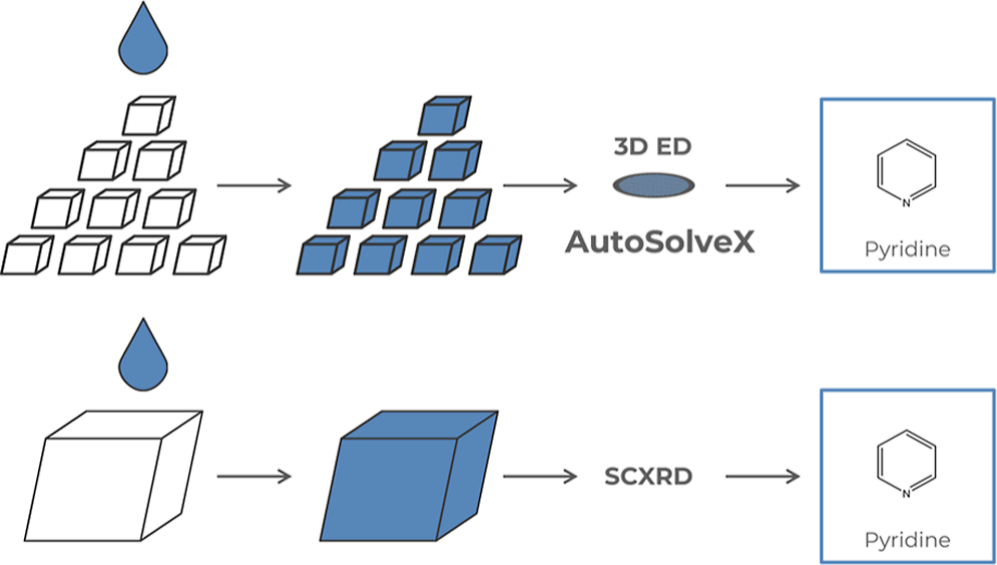
Schematic comparison of crystalline sponge (CS) protocols using SCXRD and 3D ED with automated guest identification by AutoSolveX.^34^

## Methods

### Synthesis of SU-100

As a model system, we selected the bismuth-based MOF Bi­(BPT)·2MeOH (SU-100, BPT = biphenyl-3,4′,5-tricarboxylate), which crystallizes as plate-like nanocrystals.^30^ SU-100 was synthesized under solvothermal conditions from Bi­(NO_3_)_3_·5H_2_O and H_3_BPT in methanol at 160 °C for 1 h, following our previously reported procedure.^30^ SU-100 has a flexible framework (space group *I*2/*a*), in which the Bi–O inorganic building units can bend and rotate, allowing the pore geometry to adapt. The 5 Å × 5 Å pore opening determined by 3D ED accommodates various solvent molecules with unit-cell expansion of up to 10.2%. Importantly, SU-100 is stable in air and compatible with most solvents, addressing the key limitations of earlier crystalline sponges.

### Guest-Soaking

Because methanol exhibits a weak affinity to the SU-100 framework (Figure S1), the as-synthesized material required no solvent-exchange step prior to guest soaking. We selected 10 representative guest molecules spanning different physical states and functionalities: liquids (*N*,*N*-dimetylformamide (**1**, DMF), *N*, *N*-diethylformamide (**2**, DEF), pyridine (**3**), cyclohexane (**4**)), solution-based guests (urea (**5**), 2-methylimidazole (**6**, Hmim), benzoic acid (**7**)), and vapors (ethyl acetate (**8**), benzaldehyde (**9**), isovaleraldehyde (**10**)) (Figure 2a). These molecules were chosen to cover a wide range of sizes, polarities, and interaction motifs, enabling a systematic evaluation of the nanoCS method.

**Figure 2:**
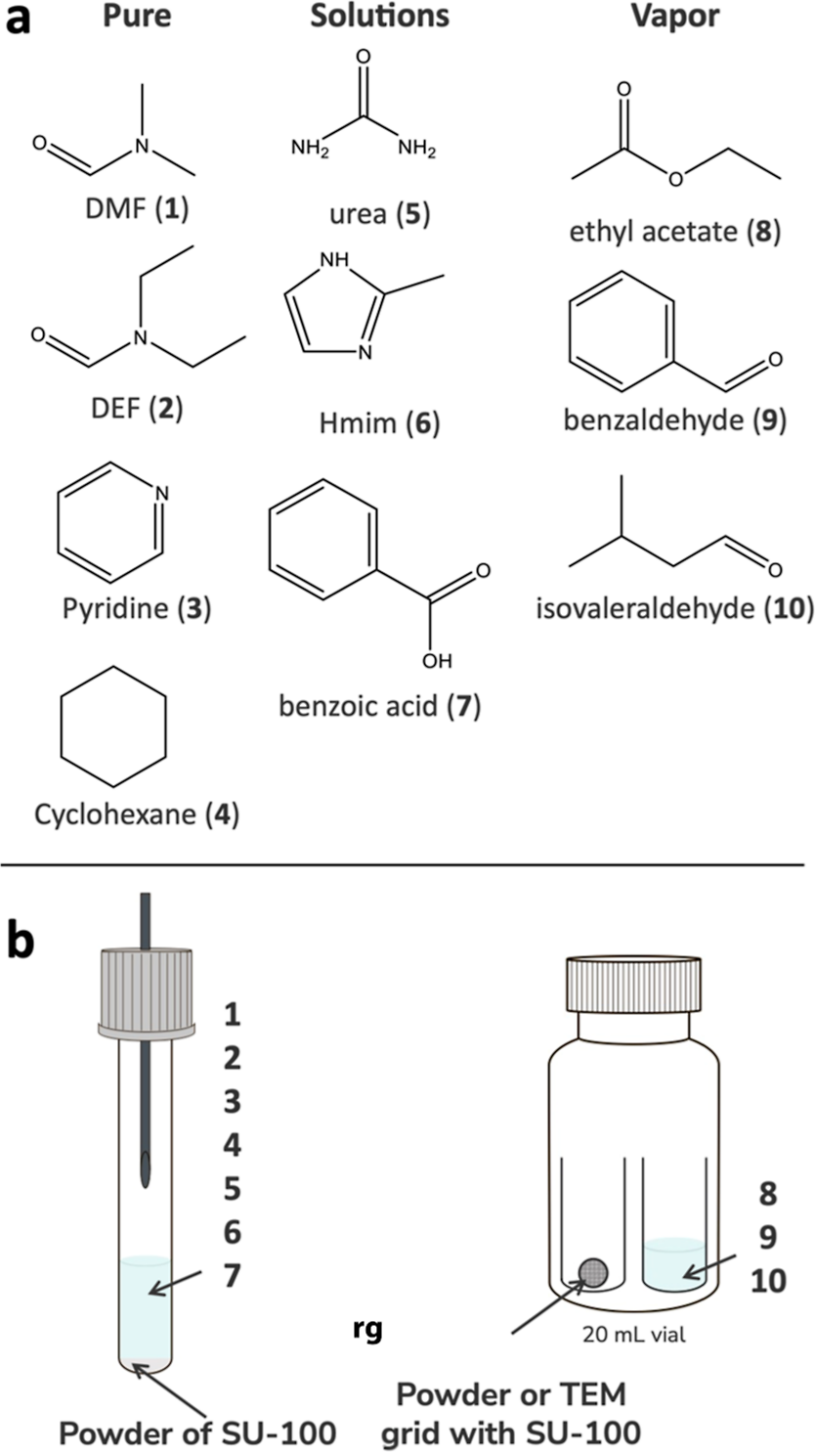
Guest-soaking setups and studied compounds. Compounds **1** and **2** were soaked under autogenous pressure; for compounds **3–7**, a needle was inserted into the vial cap to allow slow evaporation of the solvent.

Two guest-soaking strategies were employed depending on the guests: (i) liquid phase or solution-based soaking and (ii) vapor-phase soaking (Figure 2b).

#### Liquid Phase and Solution-Based Soaking (Compounds **1–7**)

Soaking was conducted under stirring at an elevated temperature. Compounds **1–2** were soaked at 100 °C for 1 h in sealed vials under autogenous pressure. Compounds **3–7** were soaked at 50 °C for 24 h under ambient pressure. For solid guests (**5–7**), methanol solutions (20 mg/mL) were prepared before soaking to ensure uniform concentrations. The MOF concentrations were 5 mg/mL for compounds **1–2** and **5–7** and 1 mg/mL for compounds **3–4**. After being soaked, the powders were filtered and dried in air.

#### Vapor-Phase Guest-Soaking (Compounds **8–10**)

Soaking was performed at room temperature via vapor diffusion onto dry SU-100 powder, a setup developed for volatile compounds.^31^ Two separate vials, one containing 15 mg of SU-100 and the other containing 0.5 mL of guest, were sealed together inside a 20 mL vial (Figure 2b). Ethyl acetate (**9**) and isovaleraldehyde (**10**) required 24 h, whereas benzaldehyde (**8**), with its higher boiling point (178 °C), required 7 days. Vapor-phase soaking was also successfully performed directly on SU-100 loaded onto TEM grids, minimizing sample consumption and simplifying the guest-soaking procedure. The vapor-trapping procedure was previously applied to [(ZnI_2_)_3_(tpt)_2_],^28^ which required *trans*-decalin as a stabilizer because of the desolvation in air.^31^ In contrast, SU-100 is inherently air-stable and can be used directly for vapor guest-soaking without any added stabilizing agents. For detailed guest-soaking procedures, see Supporting Information.

The unit cell parameters of the as-synthesized and 10 guest@SU-100 MOFs were refined against powder-X-ray diffraction data using a Pawley fit (Figures S2 and S3). The presence of guest molecules in SU-100 is confirmed by FTIR (Figures S4 and S5). Crystallographic data and refinement details for the Pawley fit are provided in Table S1. More details are presented in Supporting Information.

### 3D Electron Diffraction Data Collection

To minimize guest desorption and electron beam damage, data collection was performed under low-dose and cryogenic conditions. Dry SU-100 powders (before or after guest soaking) were gently crushed and transferred onto copper EM grids. The grids were plunge-frozen in liquid ethane and loaded into a Titan Krios G3i Cryo-TEM instrument operated at 300 kV.

3D ED data collection was performed at 80 K using a Ceta-D CMOS detector and EPU-D software (Thermo Fisher Scientific). Two data-collection conditions with different electron fluxes were employed. For compounds **1–7**, microprobe mode with extremely low flux (0.0025 e^–^/Å^2^/s) and total fluence (1 e^–^/Å^2^ per 120° rotation data set) was used to minimize radiation damage. For compounds **8–10**, nanobeam mode with ∼14-fold higher flux (0.0349 e^–^/Å^2^/s) and 5.24 times higher total fluence (5.24 e^–^/Å^2^ per 120° rotation data set) was applied. Details are given in Table S2. The 3D reciprocal lattices and 2D slices of as-synthesized SU-100 and DEF@SU-100 are shown in Figure S6.

Multiple data sets from each sample were collected and processed using XDS software.^32,33^ Because individual nanocrystals often give incomplete diffraction coverage, data sets from 2 to 3 crystals were merged to improve completeness.

### Automated Structure Solution and Guest Identification Using AutoSolveX

Guest identification from 3D ED data is typically performed by manual inspection of residual electrostatic potential (Q-peaks), a process that requires significant crystallographic expertise and can be challenging for nonspecialists. This limits throughput, reproducibility, and broader adoption of nanocrystalline sponge analysis. To overcome these limitations, we developed an automated guest identification and refinement workflow implemented in the software AutoSolveX.^34^

AutoSolveX is a Python-based pipeline with a graphical user interface (GUI) that integrates structure solution and refinement for both the 3D ED and SCXRD data. To facilitate NanoCS applications, a dedicated module for the automated guest identification is implemented. The pipeline automates the execution of SHELXT,^35^ SHELXS,^36^ and SHELXL^37^ and incorporates a geometry-based algorithm for guest recognition. The detailed description of the underlying algorithm is provided in a separate manuscript on AutoSolveX.^34^

For each guest@SU-100, merged reflection files (HKL) were used as the input. An initial framework model was generated from the as-synthesized SU-100 structure (Figure S7a) containing only framework atoms and the coordinated water molecule. This model was refined against the merged data to produce “guest-free” residual maps, yielding lists of Q-peaks corresponding to the unmodeled electrostatic potential.

The automated guest identification module then analyzes the spatial distribution of Q-peaks and compares the extracted geometries to those of selected candidate molecules generated from the SMILES strings. The candidate molecules are ranked based on their geometric agreement with the experimental Q-peak clusters. The best-matching candidates are reported automatically together with associated root-mean-square deviation (RMSD).

Once a candidate molecule is selected, AutoSolveX initially assigns the matched Q-peaks to carbon atoms and performs automated refinement. This step yields a complete preliminary guest@SU-100 model together with refinement statistics generated without manual intervention.

Using this automated pipeline, all 10 guest molecules investigated in this study were successfully identified and refined directly from the 3D ED data. To finalize each guest@SU-100 model, manual inspection and refinement were performed. The following steps were applied.

1) Removal of spurious atoms and addition of missing atoms based on chemical knowledge.
2) Atom-type reassignment based on the chemical knowledge.
3) Application of anisotropic refinement for non-hydrogen atoms
4) Addition of hydrogen atoms based on chemical knowledge.

If necessary,

5) Application of geometric restraints (DFIX) and ADP restraints (SIMU or ISOR) or constraints (EADP) to maintain chemically reasonable bond lengths and atomic displacement parameters (ADPs).
6) Introduction of EXTI in the refinement to partially compensate for dynamical effects.

## Results and Discussion

### Automated Structure Solution

We first evaluated AutoSolveX in a one-to-one comparison mode using known guest molecules. Each merged “empty” data set was matched with its corresponding candidate molecule. AutoSolveX successfully identified and positioned all guest molecules within the SU-100. The automatically generated models (Figure 3a) have reasonable geometries and required only very few minor manual steps to complete the refinement to obtain the final structures (Figure 3c). Except for cyclohexane, atom-type reassignment (from carbon to oxygen or nitrogen) was needed for the other nine guests. Additionally, there is a need to remove extra atoms for DMF, cyclohexane, urea, and benzaldehyde and add missing atoms for benzoic acid (one atom) and ethyl acetate (two atom). The entire guest identification and refinement procedure for Pyridine@SU-100 is demonstrated in Movie S1 in the Supporting Information.

**Figure 3:**
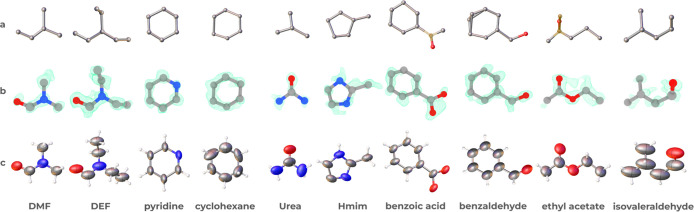
(a) Automatically obtained geometries for guests (DMF, DEF, pyridine, cyclohexane, urea, Hmim, benzoic acid, benzaldehyde, ethyl acetate, and isovaleraldehyde) using the model of as-synthesized SU-100 in AutoSolveX.^34^ (b) Difference *F*_obs_ – *F*_cacl_ maps (contoured at 0.250–0.398) showing the electrostatic potential for guests obtained after manual refinement. Molecular models are superimposed for comparison. (c) 3D structures of the guest molecules determined by nanoCS using 3D ED. The structures were refined anisotropically, and the ellipsoids are shown at 50% probability. Brown corresponds to Q-peak, gray to carbon, red to oxygen, blue to nitrogen, and white to hydrogen.

Subsequent refinement included the addition of hydrogen atoms, anisotropic refinement, application of geometric (DFIX) and ADP restraints (SIMU/ISOR or EADP), and application of EXTI in the refinement. Importantly, guest identification itself was fully automated; manual intervention was limited to refinement and polishing. Final crystallographic data and refinement details are summarized in Table S3.

### Blind Guest Screening

To evaluate guest screening capability without prior knowledge, we performed automated guest identification for each data set against all 10 candidate molecules. For each data set, AutoSolveX provides a ranking list of the best candidates based on the RMSD between the Q-peaks and candidate molecule. As shown in Table S4 and Figure S8, AutoSolveX ranked the correct molecule as No. 1 for three guests (urea, pyridine, and Hmim) and No. 2 for three guests (DMF, isovaleraldehyde, and cyclohexane). The RMSDs are almost identical to those ranked No.1 and No. 2. Three larger molecules (DEF, benzoic acid, benzaldehyde) are ranked No. 3. These molecules consist of the smaller parts, which correspond to the candidates ranked as No. 1 and 2. Ethyl acetate is ranked as No. 4, which is due to the poorly defined peak positions in the difference *F*_obs_ – *F*_cacl_ map as shown in Figure 3b.

### Discrimination of Structurally Similar Guests

Refined geometries enabled clear differentiation among closely related molecules. Cyclohexane and pyridine are readily distinguished by conformation and pore environment: cyclohexane is nonplanar and hydrophobic, whereas pyridine is planar and stabilized by N···HO­(water) hydrogen bonding (Figure S9).

Isovaleraldehyde and ethyl acetate are differentiated by geometry: the trigonal planar ester group in ethyl acetate contrasts with the predominantly tetrahedral geometry of isovaleraldehyde. Similarly, DMF and DEF are distinguished by the additional ethyl substituents in DEF, clearly resolved in the electrostatic potential maps.

Finally, in urea, the longer C–NH_2_ bond relative to C=O allows unambiguous atom assignment and confirms coordination through the carbonyl oxygen.

### Influence of Soaking Conditions on the Guest Occupancies

All non-H atoms, including those in the SU-100 framework and guest species in the pores, could be located and refined. The refined occupancies ranged from 35% for Hmim to ∼90% for benzaldehyde (Table S3). No clear correlation was observed between occupancy and the type of host–guest interaction. For example, pyridine@SU-100 (hydrogen bonding) and ethyl acetate@SU-100 (coordination bonding) both displayed ∼75% occupancy. All guest molecules could be located unambiguously, even at low occupancies and low concentrations, particularly for the vapor-phase guests, as shown by the different electrostatic potential maps.

The low occupancy of Hmim (35%) can be explained by steric incompatibility between its two crystallographic equivalent positions. The distance between the CH_3_–carbon of one equivalent and the imidazole-ring carbon of the second equivalent is 2.01(4) Å, which is too short and chemically unfeasible for simultaneous occupancy. Therefore, the site occupancy cannot exceed 50%.

Among data sets collected for the same guest species, occupancies varied by up to 10%, limiting quantitative analysis. We attribute this variation to intensity errors due to dynamical scattering and differences in guest loading across individual crystals, possibly influenced by guest-dependent diffusion kinetics. Importantly, no disorder was observed in either the host framework or guest molecules after refinement.

### Interaction between the Guest Molecules and the Framework

Differences in unit cell parameters among the guest-loaded SU-100 correlate with variations in the Bi–O coordination bond lengths and Bi–O–Bi angles within the flexible inorganic building units, consistent with previous observations by Grape et al.^30^ Across all guest-loaded structures (**1–10**), the Bi–O–Bi angle decreased from 112.9(3)° in as-synthesized SU-100 to 106.6(4)° in isovaleraldehyde@SU-100, accompanied by corresponding adjustments in Bi–O distances (Figure S10). The unit cell volume increased accordingly, from 3654(5) Å for urea@SU-100 to 3983(4)­Å for DEF@SU-100.

In as-synthesized SU-100 and in all guest-loaded structures, except cyclohexane@SU-100, benzoic acid@SU-100, and benzaldehyde@SU-100, one water molecule coordinates to each Bi center (Figure 4). The Bi–O­(water) distances range from 2.66(4) Å in the as-synthesized SU-100 to 2.78(2) Å in urea@SU-100. The SU-100 framework interacts with the guests through four main types of host–guest interactions: coordination bonding, hydrogen bonding, van der Waals interaction, and offset π–π stacking (Figure 4). Guest molecules bearing carbonyl or carboxylate groups (urea, benzoic acid, DMF, DEF, benzaldehyde, ethyl acetate, and isovaleraldehyde) coordinate to the Bi centers through their oxygen atoms (Figure 4).

**Figure 4:**
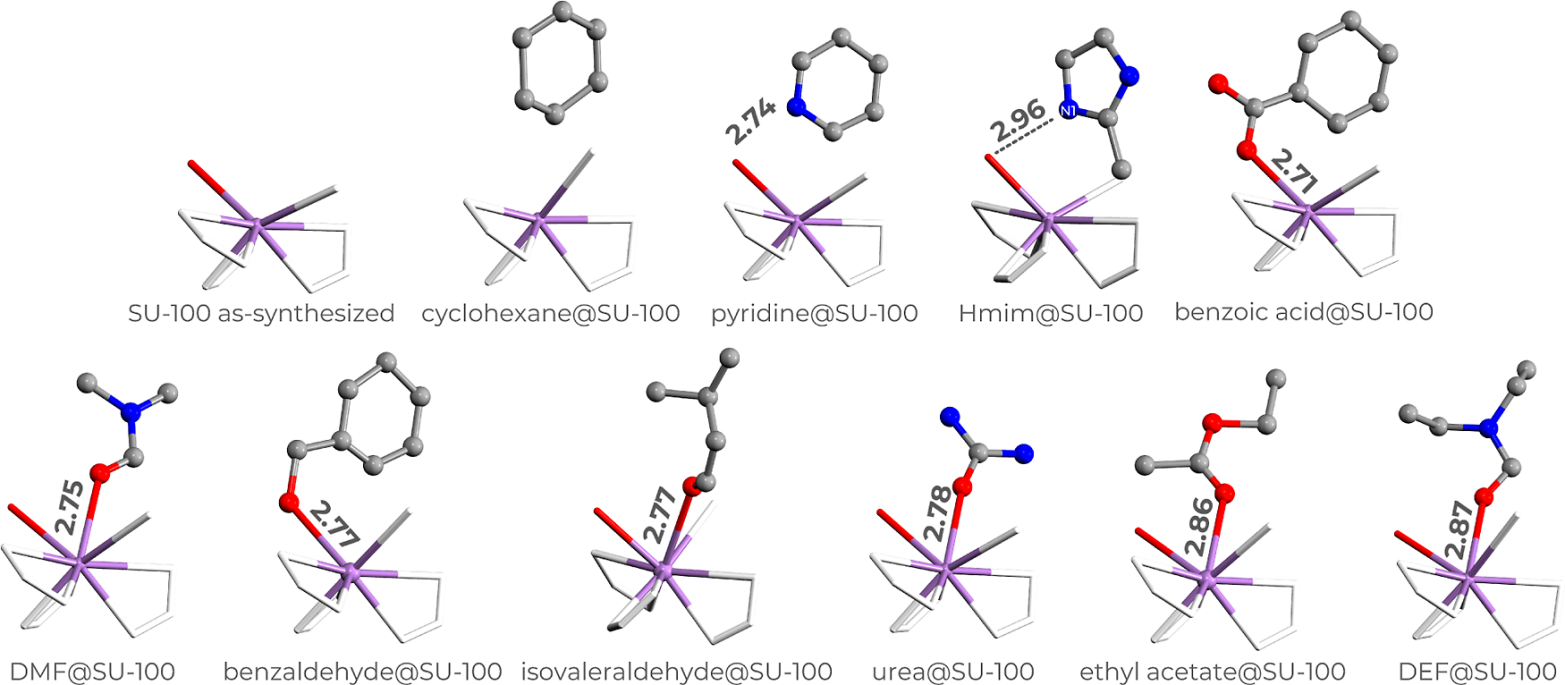
SU-100 framework with guest molecules, viewed along [001]. Bismuth atoms are shown in purple, and the oxygen atoms of the coordinated water molecule are depicted as red sticks. Marked distances correspond to host–guest Bi–O or N···HO­(water) interactions (Å). Guest molecules are shown in balls-and-stick representation.

The Bi–O­(guest) distances fall within the expected range for Bi-carboxylate and Bi-oxo coordination (2.4–2.86 Å).^38,39^ Specifically, urea binds 2.78(3) Å, benzoic acid 2.71(3) Å, DMF 2.75(2) Å, DEF 2.87(3) Å, benzaldehyde 2.77(2) Å, ethyl acetate 2.86(4) Å, and isovaleraldehyde 2.77(5) Å (Figure 4).

Hmim and pyridine interact with the SU-100 host framework through hydrogen bonding rather than coordination. Hmim forms a N1···HO­(water) hydrogen bond of 2.96(3) Å, whereas pyridine forms a N···HO­(water) interaction of 2.74(3) Å (Figure 4).

Despite lacking functional groups, cyclohexane is ordered within the pores through van der Waals interactions (Figure 4). Hirschfeld surface analysis^40^ (Figure S11) confirms the tight packing of cyclohexane molecules, facilitated by the relatively small pore dimensions of SU-100.

Aromatic guests, benzoic acid, Hmim, pyridine, and benzaldehyde, also participate in offset-type π–π stacking with neighboring molecules or with the BPT linker. The shortest distances between the aromatic ring centroid of the guest and the interacting hydrogen atom in the framework ring are 3.2(1) Å for benzoic acid, 3.2(1) Å for pyridine, and 3.6(1) Å for benzaldehyde. For Hmim, the imidazole hydrogen interacts with the linker’s benzene ring at a distance of 3.0(1) Å (Figure S12).

Overall, we observed a clear trend: guest molecules containing carbonyl or carboxylate groups preferentially coordinate to the Bi centers, whereas guests possessing only pyridinic nitrogen form hydrogen bonds and nonpolar molecules rely on van der Waals interactions. These distinctions reflect how a MOF adapts its local environment to accommodate guests of varying polarities and geometry.

### Suggestions for the Design of a Suitable CS for 3D ED

Compared with SCXRD-based crystalline sponge methods, the nanoCS approach offers greater flexibility in both the guest-soaking procedures and data-collection conditions. Unlike the Zn-MOF used in the original method by Fujita’s group,^1^ SU-100 can be used directly after synthesis: its synthesis solvent (MeOH) has low affinity for the framework, eliminating the need for solvent exchange. Guest molecules can therefore be soaked directly in the dry as-synthesized powder. Soaking typically required only 1 day for compounds **1–9**, compound **10** required a longer time due to its higher boiling point, though we expect that soaking pure liquid guest would require less time.

3D ED data-collection parameters can be adjusted without compromising structural quality. Importantly, guest occupancies remained consistent across samples prepared at different concentrations, demonstrating that the method is broadly tolerant to variations in concentrations and loading. Cryogenic conditions help mitigate beam damage, and electron fluence should be optimized for each CS system because beam sensitivity differs among frameworks and guest types. For guest molecules coordinated to the metal centers, such as for **8–10**, higher electron fluence may be applied to increase the signal-to-noise ratio and improve the data quality.

Smaller pore sizes can be advantageous when the target molecules are small and weakly interacting. In SU-100, the relatively narrow pores were able to align inert cyclohexane molecules through enhanced van der Waals interactions, whereas cyclohexane was disordered in the larger-pore Zn-MOF used in Fujitás studies.^9^ Stronger confinement can therefore improve guest ordering and reduce disorders.

A suitable CS material for 3D ED should be stable under vacuum and under electron irradiation, and for practical handling, stability in air is also desired. Structural flexibility is another beneficial feature: flexible MOFs can accommodate a wider range of guest geometries without losing crystallinity, and their stability in air allows monitoring of guest uptake by PXRD. Meeting these criteria will expand the number of MOFs suitable for nanoCS applications.

Finally, we propose that the significantly smaller crystal sizes used in 3D ED both accelerate guest diffusion and reduce the minimum sample amount required for structure determination compared to conventional SCXRD. The shorter diffusion paths in nanocrystals also contribute to faster soaking times, as observed for SU-100.

## Conclusions

We demonstrate that 3D electron diffraction (3D ED) enables ab initio atomic structure determination of guest molecules within a crystalline sponge and supports a simplified crystalline sponge (CS) workflow that eliminates the need for large single crystals. Using the flexible bismuth-based MOF SU-100 as a prototype nanocrystalline sponge (NanoCS), we determined the structures of 10 small organic molecules introduced as liquids, solutions, or vapors. By employing a low-affinity solvent in the synthesis, we can use SU-100 directly after synthesis without activation or solvent exchange. Guest soaking was straightforward and typically completed within 1 day for compounds **1–9**, with longer soaking required only for the higher-boiling compound **10**.

A key methodological advance of this work is the integration of automated guest identification and refinement using AutoSolveX. All 10 guest molecules were successfully identified and positioned directly from 3D ED data, and blind cross-screening demonstrated a reliable automated ranking of candidate guests. Manual intervention was limited to refinement polishing rather than guest localization, establishing NanoCS as a high-throughput, largely unbiased platform for structure determination.

Using cryogenic 3D ED, all non-hydrogen atoms of both framework and guests were located and refined. Guest occupancies ranged from 35% to ∼90%, yet even low-occupancy guests were unambiguously resolved in electrostatic potential maps. Framework flexibility is reflected in systematic structural adaptation upon guest incorporation: the Bi–O–Bi angle decreases, and the unit cell expands from 3617(9) Å^3^ for as-synthesized SU-100 to 3961(3) Å^3^ for DEF@SU-100. This expansion enables convenient monitoring of guest uptake by PXRD.

The incorporated guests exhibit four principal interaction modes with the framework: coordination bonding, hydrogen bonding, van der Waals interactions, and offset π–π stacking. Carbonyl- and carboxylate-containing molecules preferentially coordinate to Bi centers, pyridinic guests engage in hydrogen bonding, and nonpolar molecules are stabilized through confinement-driven van der Waals interactions. These trends highlight how a flexible MOF adapts its local environment to accommodate guests of varying polarities and geometry.

Together, these results define key criteria for 3D ED-compatible crystalline sponges: air and vacuum stability, resistance to electron irradiation, structural flexibility, and pore dimensions that promote guest ordering. By combining nanocrystalline hosts with automated structural analysis, the new NanoCS workflow expands the scope of the crystalline sponge method and establishes a practical route for the routine ab initio structure determination of organic molecules from nanocrystalline materials.

## Supplementary Material





## Notes

Entire guest identification and refinement procedure for Pyridine@SU-100 (MP4)
Experimental details, structure determination of as-synthesized SU-100 by 3D ED, guest identification and refinement, crystallographic details for 3D ED, Pawley fits, FTIR data, and Hirschfeld surface analysis for visualization of host–guest interactions and offset π–π stacking interaction visualization (PDF)

## †

J.C.—California NanoSystems Institute, University of California, Los Angeles, California 90095, USA
