# Supplementary material for: Automated Nanocrystalline Sponge Workflow Enabled by 3D Electron Diffraction
Source: J Am Chem Soc. 2026 Mar 5;148(10):11081–8. doi: 10.1021/jacs.5c21773 (PMC13003498; doi:10.1021/jacs.5c21773)
Supplement: Supplementary file 2 [file ja5c21773_si_002.pdf]

SUPPORTING INFORMATION

Automated Nano-Crystalline Sponge Workflow Enabled by 3D Electron Diffraction

Sofiia Butonova,[a] Yinlin Chen,[a] Jung Cho,*[a],† Marcus Wallin,[a] Zhehao Huang,[a] Xiaodong Zou*[a].

[a] Department of Chemistry, Stockholm University, SE-106 91, Stockholm, Sweden.

† Present Addresses: California NanoSystems Institute, University of California, Los Angeles, California 90095, USA.

Contents

Materials and methods 3
Chemicals. 3
Guest-soaking. 3
Characterization 3
Screening guest inclusion by PXRD and FTIR 3
Structure determination of as-synthesized SU-100 by 3D ED 4
Guest identification and refinement 4
Table S1. Crystallographic data and refinement details for the Pawley fit. 6
Table S2. 3D ED data collection conditions for as-synthesized SU-100 and guest@SU-100. 6
Table S3. Crystallographic table for electron diffraction data of as-synthesized SU-100 and SU-100 after soaking of guest molecules. Anisotropic refinement. In brackets: state of the guest molecule. 7
Table S4. Guest identification ranking by AutoSolveX. 10
Figure S1. FTIR spectra under the vacuum for as-synthesized SU-100. 12
Figure S2. Pawley fit of the experimental PXRD pattern (λ = 1.5406 Å) of as-synthesized SU-100 and SU-100 after guest soaking as pure phase and in solution. 13
Figure S3. Pawley fit of the experimental PXRD pattern (λ = 1.5406 Å) of SU-100 after soaking vapor molecules. Additional crystals corresponding to benzaldehyde (blue pattern) were found in the vial and on the vial's walls with benzaldehyde@SU-100 (black pattern). Pawley fit for benzaldehyde@SU-100 was performed, excluding peaks corresponding to pure benzaldehyde. Intensities are normalized to the benzaldehyde@SU-100 plot. 14
Figure S4. FTIR spectrum of SU-100 after guest soaking of pure phase and in solution. 15
Figure S5. FTIR spectrum of SU-100 after guest soaking as vapors. 16
Figure S6. (a) 3D reciprocal lattice of as-synthesized SU-100. (b-d) 2D slices cut from the reconstructed 3D reciprocal lattice of SU-100 showing (b) 0*kl*; (c) *hk*0; (d) *h*0*l*. (e) 3D reciprocal lattice of DEF@SU-100. (f) 0*kl*; (g) *hk*0; (h) *h*0*l*. 17
Figure S7. (a) The structure of as-synthesized SU-100 viewed along [010]. Bismuth, carbon, and oxygen atoms are depicted in purple, grey, and red, respectively; (b) PXRD patterns of as-synthesized SU-100 and SU-100 after soaking different guest molecules. The intensities are normalized. 17
Figure S8. Guest identification ranking by AutoSolveX. 18
Figure S9. Refined geometries of cyclohexane and pyridine in SU-100. 18
Figure S10. Framework pore structures of as-synthesized SU-100 and SU-100 after soaking of guest molecules, ... 19
Figure S11. Structures of as-synthesized SU-100 and guest@SU-100, viewed along [010] showing the Hirshfeld surface of the guest molecules and their locations at the intersection of the pores of SU-100. 19
Figure S12. Offset-type π- π stacking interactions between aromatic guests and the SU-100 framework. 20
References 21

Materials and methods

Chemicals. Bismuth(III) nitrate pentahydrate (98%), biphenyl-3,4′,5-tricarboxylic acid (H_3BPT) (96%), 2-methylimidazole (Hmim), Benzoic acid (≥99.5%), cyclohexane (anhydrous, 99.5%), methanol, N,N-Dimethylformamide (99.5%), isovaleraldehyde (97%), were purchased from Sigma-Aldrich. Urea was purchased from Alfa Aesar. Pyridine and ethyl acetate (99.9%) were purchased from VWR. *N*,*N*-Diethylformamide (>99.0) was purchased from TCI. Benzaldehyde (≥98%) was purchased from ACROS Organics. All chemicals were used without further purification.

Guest-soaking. The guest soaking was conducted both at room temperature from vapor (compound **8-10**, Figure 2b) and at elevated temperatures (50 or 100 °C) in an Al heating block from liquid/solution (compound **1-7**, Figure 2b) under stirring. For DMF (**1**) and DEF (**2**), 3 mL of the compound was mixed together with 15 mg of SU-100 and heated at 100°C for 1 h under autogenous pressure, following the procedure reported by Grape *et al.*[1] For pyridine (**3**) and cyclohexane (**4**), 4 mL of the compound was dropped onto 4 mg of as-synthesized SU-100, followed by heating at 50°C for 24 h at ambient pressure. For solids, urea (**5**), Hmim (**6**), and benzoic acid (**7**), 80 mg of the compound was first dissolved in 4 mL of methanol, after which 20 mg of as-synthesized SU-100 was added to the solution. The mixture was heated at 50 °C for 24 h under stirring at ambient pressure (Figure 2b).[2] For those in vapor phase, **8-10,** the setup developed for volatile compounds was used.[3] Two small vials, one with 15 mg of as-synthesized SU-100 and the other with 0.5 mL of the compound, were sealed in a 20 ml vial (Figure 2b). The soaking was conducted at room temperature via the vapor diffusion of the volatile compound. The soaking time was 24 h for ethyl acetate (**9**) and isovaleraldehyde (**10**), but much longer, 7 days, for benzaldehyde (**8**) attributed to its higher boiling temperature (178 °C), or lower vapor pressure (133 Pa) compared to those of others.

Characterization

Powder X-ray diffraction (PXRD) was performed using Bruker D8 DISCOVER diffractometer with $Cu_{K\alpha}$ radiation ($\lambda_{CuK\alpha1}$ = 1.5406 Å) with 2θ range 5 – 40° and step size 0.005°. Powder X-ray diffraction data were indexed and Pawley fit was done using TOPAS-Academic V6 software.[4] Fourier-transform infrared (FTIR) spectra were recorded using an ATR mode with a Varian 670-IR spectrometer.

Screening guest inclusion by PXRD and FTIR

Changes in the unit cell parameters of the as-synthesized SU-100 were observed by adsorbing DEF and DMF.[1] This property of the MOF enables the use of powder X-ray diffraction (PXRD) as a simple and effective tool to monitor the progress of guest adsorption. PXRD patterns (Figure S7b) collected after the inclusion of the guest molecules demonstrated volume expansion, indicating the presence of guest molecules inside the pores. Volume expansion ranged from 3617(9) Å for as-synthesized SU-100 to 3961(3) Å for DEF@SU-100. The crystallographic details of as-synthesized SU-100, and guest@SU-100 (**1-10**) from Pawley fits against the PXRD data are summarized in Figure S2 and S3. For 3D ED, we are limited to the number of crystals picked up for the data collection. We complement our analysis with PXRD to acquire data representative of bulk samples. Then, the solvent exchange process from methanol was monitored using IR spectroscopy (Figures S4 and S5). To characterize the guest molecules within the pores, 3D ED data collection was performed.

Structure determination of as-synthesized SU-100 by 3D ED

3D ED data collection was carried at 80 K on Titan Krios G3i Cryo-TEM operated at 300 kV using a Ceta-D CMOS detector and the EPU-D software (Thermo Fisher Scientific). A parallel beam was generated with a combination of C2 and C3 condenser lenses using a 20 μm C2 aperture with the spot size 10. Two different data collection conditions were tested. Data collection for compounds **1-7** was conducted in microprobe mode with a low flux (0.0025 $e^-/Å^2/s$). The rotation speed of the goniometer was 0.3°/s with the exposure time of 1 s/frame. The total fluence for each dataset with a 120° rotation range was 1 $e^-/Å^2$. For compounds **8-10**, the data was collected in nanobeam mode with a 14 times higher flux of 0.0349 $e^-/Å^2/s$, spot size 11. The rotation speed of the goniometer was faster (0.8°/s) and the exposure time was reduced accordingly to 0.25 s/frame. The total fluence for each dataset with a 120° rotation was increased to 5.24 $e^-/Å^2$, 5.24 times higher compared to that for compounds **1-7**. Details are given in Table S2.

Unit cell parameters for as-synthesized SU-100 determined by 3D ED were a = 20.897(11), b = 10.000(8), c = 17.850(8) Å, β = 96.98(4)°. Based on the 2D slices cut from the 3D reciprocal lattice (Figure S6), the reflection conditions were deduced to be hkl: $h + k + l = 2n$, $0kl$: $k + l = 2n$, $hk0$: $h + k = 2n$, $h0l$: $h, l = 2n$, $0k0$: $k = 2n$, consistent with two space groups: *Ia* (no. 9) and *I*2/*a* (no. 15). The as-synthesized SU-100 structure has been solved and refined from the 3D ED data using the space group *I*2/*a* (no. 15), in agreement with the published structure (Figure S6 a-d). [1] The inorganic building unit of SU-100 consists of Bi^{3+} with a coordination number of 7, with two oxygen atoms bridging the two Bi^{3+} in an edge-sharing manner and one water molecule coordinating to each Bi center (Figure S7a). To locate guest molecules, an automated guest screening and refinement was applied using AutoSolveX[5], followed by manual inspection and refinement. The structure shows no methanol inside the pores. This observation is consistent with the FTIR study, which demonstrates the desorption of methanol from SU-100 at 10^{-2}–10^{-3} Torr (Figure S1), indicating that methanol was removed from the pores of SU-100 under high vacuum inside the TEM column.

Guest identification and refinement

An automated workflow for guest identification and refinement workflow was developed and implemented in the software AutoSolveX.[5] For each guest@SU-100 dataset, a merged 3D ED intensity HKL file was used as input. The same guest-free SHELX INS file, containing only the framework atoms of SU-100 including one coordinated water molecule (Figure S7a), was used for guest identification in all ten guest@SU-100 datasets. This INS file was generated from the final RES file obtained after refinement of the as-synthesized SU-100 structure.

To generate guest-free ("empty") RES files containing Q-peaks for guest identification, the framework-only model was refined against each merged HKL file (guest@SU-100) using AutoSolveX. All framework atoms were refined isotopically without restraints. The number of Q-peaks (PLAN) was set to 20. The resulting Q-peak lists were then used for automated guest identification by comparing their geometries with those of selected candidate molecules implemented in AutoSolveX. The results are summarized in Table S4 and Figure S8.

The guest identification algorithm is described in the corresponding publication.[5] Briefly, for each host–guest dataset, Q-peaks located too close to the framework atoms are first removed. The remaining Q-peaks are expanded through crystallographic symmetry operations in three-dimensional space to generate Q-peak clusters. Each candidate guest molecule is then matched to these clusters by identifying the subset of atoms that best fits the cluster geometry. The ranking is based on the root-mean-square-deviation (RMSD) estimated between the theoretical molecular

model and the experimental Q-peak cluster. After identification, the Q-peaks that best match the selected molecule are assigned as carbon atoms and subject to automated refinement within AutoSolveX. The refinement statistics are generated automatically.

After finalizing the guest@SU-100 structures, we found that the body-centered *I*2/*a* (no. 15) space group of as-synthesized SU-100 is retained after guest incorporation (Figure S6 e-h), resulting two guest molecules occupying crystallographically equivalent positions inside the pore (Figure S10). All framework atoms were determined *ab initio*. The unit cell parameters for DEF@SU-100 are comparable with the published values determined by PXRD.[1] The unit cell parameters of the other samples obtained by 3D ED agree well with those from Pawley fits (Table S3 and S1). Crystallographic details for as-synthesized SU-100 and all guest@SU-100 are summarized in Table S3.

While the framework atoms can be refined freely, soft restraints on bond distances were required for refinement of guest molecules in SU-100, which is also common for X-ray diffraction analyses. Because of dynamical scattering effects that affect diffraction intensities, the R1 values range from 0.2120 to 0.2534 (for all reflections). The standard uncertainties of guest bond distances are approximately 0.02 Å, higher than typical values for X-ray diffraction but sufficient for reliable structural assignment.

Table S1. Crystallographic data and refinement details for the Pawley fit.

		Guest in solution			Pure liquid guest				Guest in vapor		
Sample name	as-synthesized SU-100	urea@SU-100	benzoic acid@SU-100	Hmim@SU-100	pyridine@SU-100	DMF@SU-100	cyclohexane @SU-100	DEF@SU-100	benzaldehyde @SU-100	isovaleraldehyde@SU-100	ethyl acetate@SU-100
Space group	*I*2/*a* (№ 15)										
a (Å)	21.108(3)	21.039(7)	20.943(1)	20.931(2)	20.440(1)	20.871(1)	20.488(4)	20.682(1)	20.258(12)	20.389(6)	21.035(2)
b (Å)	9.627(1)	9.801(3)	9.860(5)	9.872(1)	10.488(1)	10.258(1)	10.785(2)	10.760(1)	10.829(6)	10.903(4)	10.135(1)
c (Å)	17.926(2)	17.867(6)	17.836(8)	17.859(2)	17.756(1)	17.827(1)	17.879(3)	18.038(1)	17.884(11)	18.010(6)	17.737(3)
β (°)	96.798(1)	97.211(1)	96.864(2)	97.011(1)	96.055(8)	97.508(3)	98.930(1)	99.335(4)	97.282(5)	98.181(7)	97.074(11)
V (Å^3)	3617.2(9)	3655.2(2)	3656.8(3)	3662.4(6)	3785.4(5)	3784.1(2)	3902.8(1)	3961.0(3)	3891.5(40)	3962.8(2)	3752.6(8)
R_{wp}	0.05	0.09	0.11	0.09	0.11	0.09	0.10	0.09	0.13	0.11	0.09

Table S2. 3D ED data collection conditions for as-synthesized SU-100 and guest@SU-100.

Data Collection		
	pure and in solutions	vapors
Camera	Ceta-D, CMOS detector	
Temperature (K)	80(2)	
Radiation, wavelength (Å)	Electrons, 0.0197	
Electron dose rate, flux ($e^-/Å^2 \cdot s$)	0.0025	0.0349
Tilt step per frame (°)	0.3	0.8
Exposure time per frame (s)	1	0.25
Spot size	10	11
Tilt range (°)*	90 – 130	80 – 130
Total fluence for 120° ($e^-/Å^2$)	1	5.24

*Tilt range: minimum angle for individual dataset – maximum angle for individual dataset

Datasets were merged without applying a resolution cut-off, and the resolution was then cut for a merged dataset in XDSCONV step. The resolution cut-off was applied in XDS[6,7] by considering only data with I/SIGMA >1 and CC(1/2) with * (Correlation significant at the 0.1% level is marked by an asterisk[8]).

Table S3. Crystallographic table for electron diffraction data of as-synthesized SU-100 and SU-100 after soaking of guest molecules. Anisotropic refinement. In brackets: state of the guest molecule.

	**as-synthesized SU-100**	**urea@SU-100 (solution)**	**Hmim@SU-100 (solution)**	**benzoic acid@SU-100 (solution)**
Number of datasets	3	2	2	3
Resolution (Å)	0.74	0.77	0.78	0.78
Crystal system	monoclinic			
Space group	*I*2/*a* (15)			
a (Å)	20.897(11)	21.342(23)	21.128(40)	21.166(15)
b (Å)	10.000(8)	9.600(8)	9.780(12)	9.8000(12)
c (Å)	17.850(8)	17.950(8)	17.870(12)	17.970(7)
β (Å)	96.98(4)	96.49(5)	96.31(4)	96.57(3)
Volume ($Å^3$)	3702(4)	3654(5)	3670(9)	3703(3)
**Data Collection**				
Reflections collected	33038	18358	20428	26165
R_{int}	0.2569	0.1985	0.2344	0.2920
Completeness (%)	95.4	95.7	99.3	95.5
**Refinement**				
$N_{\text{Independent reflections}}$	4564	3936	3987	3846
$N_{\text{restraints}}$	6	17	7	6
$N_{\text{parameters}}$	197	188	236	235
R_1 [I>=2σ (I)]	0.2120	0.1659	0.2016	0.1621
R_1 [all data]	0.2415	0.2182	0.2282	0.2325
wR_2 [all data]	0.4948	0.4333	0.4598	0.4468
GooF	1.151	1.141	1.177	1.122
Occupancy of the guest molecule, ~ (%)	0	65	35	45

Table **S3** continue

	**ethyl acetate@SU-100 (vapor)**	**DMF@SU-100 (pure)**	**pyridine@SU-100 (pure)**	**benzaldehyde@SU-100 (vapor)**
Number of datasets	2	2	2	2
Resolution (Å)	0.87	0.72	0.76	0.78
Crystal system	monoclinic			
Space group	*I*2/*a* (15)			
a (Å)	20.886(29)	21.320(28)	21.024(101)	20.416(41)
b (Å)	10.130(11)	9.880(5)	10.040(17)	10.720(14)
c (Å)	17.730(5)	17.830(22)	17.840(24)	17.890(11)
β (Å)	96.55(3)	96.82(9)	96.05(12)	96.65(4)
Volume (Å^3)	3727(7)	3729(7)	3745(19)	3889(10)
**Data Collection**				
Reflections collected	13920	20741	21816	21871
R_{int}	0.2985	0.2016	0.2296	0.2129
Completeness (%)	99.1	96.0	99.6	97.7
**Refinement**				
$N_{\text{Independent reflections}}$	2938	4447	4292	4193
$N_{\text{restraints}}$	3	4	0	3
$N_{\text{parameters}}$	146	233	151	231
R_1 [I>=2σ (I)]	0.1591	0.2088	0.1645	0.2173
R_1 [all data]	0.2443	0.2405	0.2161	0.2450
wR_2 [all data]	0.4147	0.4923	0.4106	0.4892
GooF	1.111	1.131	1.148	1.245
Occupancy of the guest molecule, ~ (%)	75	60	75	90

Table **S3** continue

	isovaleraldehyde@SU-100 (vapor)	cyclohexane@SU-100 (pure)	DEF@SU-100 (pure)
Number of datasets	2	2	2
Resolution (Å)	0.78	0.80	0.77
Crystal system	monoclinic		
Space group	*I*2/*a* (15)		
a (Å)	20.291(38)	20.503(39)	20.932(14)
b (Å)	10.860(11)	10.810(10)	10.640(6)
c (Å)	18.000(19)	17.960(29)	18.110(7)
β (Å)	97.52(8)	98.38(13)	99.06(3)
Volume (Å^3)	3932(9)	3938(11)	3983(4)
**Data Collection**			
Reflections collected	21070	20900	19275
R_{int}	0.3138	0.2163	0.1720
Completeness (%)	99.1	91.2	90.6
**Refinement**			
$N_{\text{Independent reflections}}$	4300	3677	4003
$N_{\text{restraints}}$	5	3	7
$N_{\text{parameters}}$	236	255	239
R_1 [I>=2σ (I)]	0.2149	0.2046	0.1690
R_1 [all data]	0.2534	0.2227	0.2120
wR_2 [all data]	0.4918	0.4634	0.4430
GooF	1.092	1.167	1.115
Occupancy of the guest molecule, ~ (%)	70	75	80

Table S4. Outcome of the guest identification and ranking by AutoSolveX[5]. The ranking is based on the root mean square deviation (RMSD). Those corresponding to the true guest molecules are marked in green. The RMSDs are within 0.20 Å for all true molecule candidates except for Hmim and ethyl acetate.

Dataset	Guest candidate	Scale	RMSD (Å)	Nq	Nmol	Cluster No	Q-peak label	Ranking
urea@SU-100	urea	1.02	0.08	5	4	5	2, 4, 5, 6, 7	1
	ethyl acetate	1.08	0.20	6	6	5	2, 4, 5, 6, 7, 8	2
	isovaleraldehyde	1.04	0.22	6	6	5	2, 4, 5, 6, 7, 8	3
	DEF	1.10	0.27	7	7	5	2, 4, 5, 6, 7, 8, 9	4
	DMF	0.89	0.28	5	5	5	2, 4, 5, 6, 7	5
pyridine@SU-100	pyridine	1.02	0.03	6	6	2	3, 4, 5, 6, 7, 8	1
	cyclohexane	1.07	0.06	6	6	2	3, 4, 5, 6, 7, 8	2
	benzoic acid	1.11	0.24	9	9	2	3, 4, 5, 6, 7, 8, 9, 10, 12	3
	benzaldehyde	1.13	0.27	8	8	2	3, 4, 5, 6, 7, 8, 9, 10	4
isovaleraldehyde@SU-100	urea	0.96	0.11	4	4	3	3, 4, 5, 6	1
	isovaleraldehyde	1.05	0.13	6	6	3	3, 4, 5, 6, 7, 8	2
	DMF	1.00	0.20	5	5	3	3, 4, 5, 6, 7	3
	ethyl acetate	1.09	0.21	6	6	3	3, 4, 5, 6, 7, 8	4
	Hmim	0.87	0.23	6	6	3	3, 4, 5, 6, 7, 8	5
	cyclohexane	1.18	0.28	7	6	3	3, 4, 5, 6, 7, 8, 9	6
	pyridine	1.12	0.28	7	6	3	3, 4, 5, 6, 7, 8, 9	7
	benzaldehyde	1.23	0.32	8	8	3	3, 4, 5, 6, 7, 8, 9, 10	8
	DEF	1.16	0.32	7	7	3	3, 4, 5, 6, 7, 8, 9	9
	benzoic acid	1.34	0.32	9	9	3	3, 4, 5, 6, 7, 8, 9, 10, 12	10
Hmim@SU-100	Hmim	1.13	0.29	7	6	3	3, 3, 4, 5, 6, 7, 8	1
ethyl acetate@SU-100	DEF	0.89	0.21	9	7	5	2, 3, 5, 6, 8, 10, 13, 13, 15	1
	isovaleraldehyde	0.91	0.25	7	6	5	2, 3, 5, 6, 8, 10, 13	2
	DMF	0.99	0.26	6	5	5	2, 3, 5, 6, 8, 10	3
	ethyl acetate	0.84	0.28	6	6	5	2, 3, 5, 6, 8, 10	4
	urea	1.13	0.31	6	4	5	2, 3, 5, 6, 8, 10	5

Table S4 continue

Dataset	Guest candidate	Scale	RMSD (Å)	Nq	Nmol	Cluster No	Q-peak label	Ranking
DMF@SU-100	urea	0.98	0.13	4	4	0	2, 3, 5, 6	1
	DMF	1.11	0.13	6	5	0	2, 3, 5, 6, 7, 8	2
	ethyl acetate	1.03	0.16	8	6	0	2, 3, 5, 6, 7, 8, 9, 10	3
	isovaleraldehyde	1.00	0.22	8	6	0	2, 3, 5, 6, 7, 8, 9, 10	4
	DEF	0.79	0.37	8	7	0	2, 3, 5, 6, 7, 8, 9, 10	5
DEF@SU-100	DMF	1.08	0.08	5	5	2	1, 2, 3, 4, 7	1
	urea	1.00	0.17	4	4	2	1, 2, 3, 4	2
	DEF	1.02	0.19	7	7	2	1, 2, 3, 4, 7, 8, 9	3
	isovaleraldehyde	1.23	0.26	7	6	2	1, 2, 3, 4, 7, 8, 9	4
	ethyl acetate	1.16	0.36	6	6	2	1, 2, 3, 4, 7, 8	5
cyclohexane@SU-100	pyridine	0.96	0.08	6	6	3	1, 2, 3, 4, 5, 6	1
	cyclohexane	1.01	0.09	6	6	3	1, 2, 3, 4, 5, 6	2
	benzoic acid	1.33	0.47	9	9	3	1, 2, 3, 4, 5, 6, 7, 9, 10	3
	benzaldehyde	1.54	0.51	10	8	3	1, 2, 3, 4, 5, 6, 7, 9, 10, 11	4
benzoic acid@SU-100	benzaldehyde	0.92	0.17	8	8	2	3, 5, 6, 7, 8, 9, 11, 13	1
	pyridine	0.94	0.18	7	6	2	3, 5, 6, 7, 8, 9, 11	2
	benzoic acid	0.97	0.18	9	9	2	3, 5, 6, 7, 8, 9, 11, 13, 14	3
	cyclohexane	0.99	0.18	7	6	2	3, 5, 6, 7, 8, 9, 11	4
benzaldehyde@SU-100	pyridine	0.98	0.08	6	6	5	3, 4, 5, 6, 7, 9	1
	cyclohexane	1.03	0.09	6	6	5	3, 4, 5, 6, 7, 9	2
	benzaldehyde	0.95	0.20	8	8	5	3, 4, 5, 6, 7, 9, 10, 11	3
	benzoic acid	1.08	0.25	10	9	5	3, 4, 5, 6, 7, 9, 10, 11, 11, 12	4

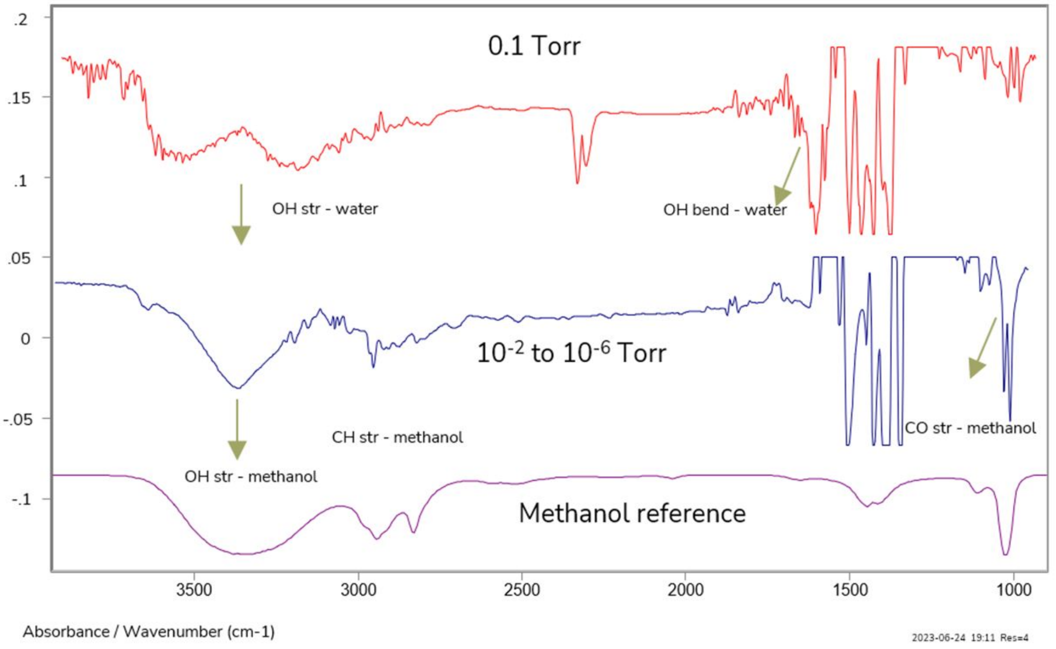

Figure S1. FTIR spectra for as-synthesized SU-100 collected under the vacuum.

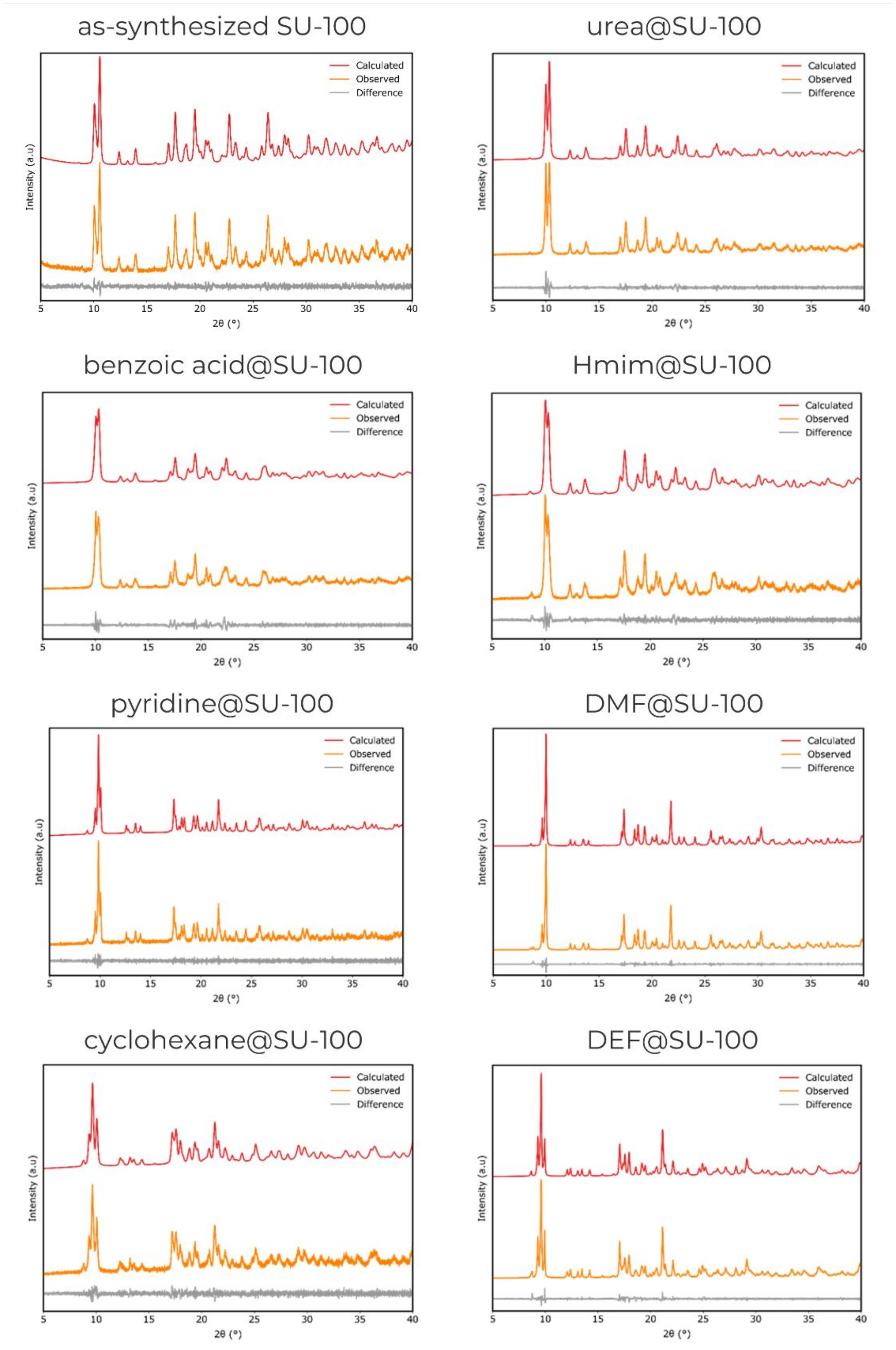

Figure S2. Pawley fit of the experimental PXRD pattern (λ = 1.5406 Å) of as-synthesized SU-100 and SU-100 after guest soaking as pure phase and in solution.

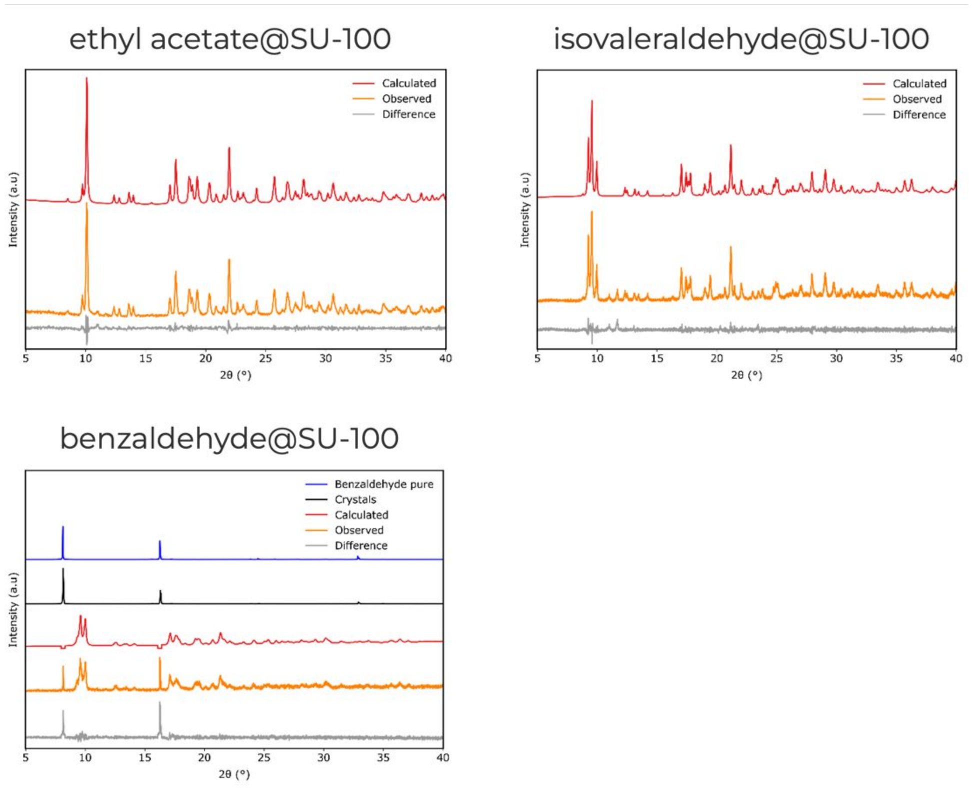

Figure S3. Pawley fit of the experimental PXRD pattern (λ = 1.5406 Å) of SU-100 after soaking vapor molecules. Additional crystals corresponding to benzaldehyde (blue pattern) were found in the vial and on the vial's walls with benzaldehyde@SU-100 (black pattern). Pawley fit for benzaldehyde@SU-100 was performed, excluding peaks corresponding to pure benzaldehyde. Intensities are normalized to the benzaldehyde@SU-100 plot.

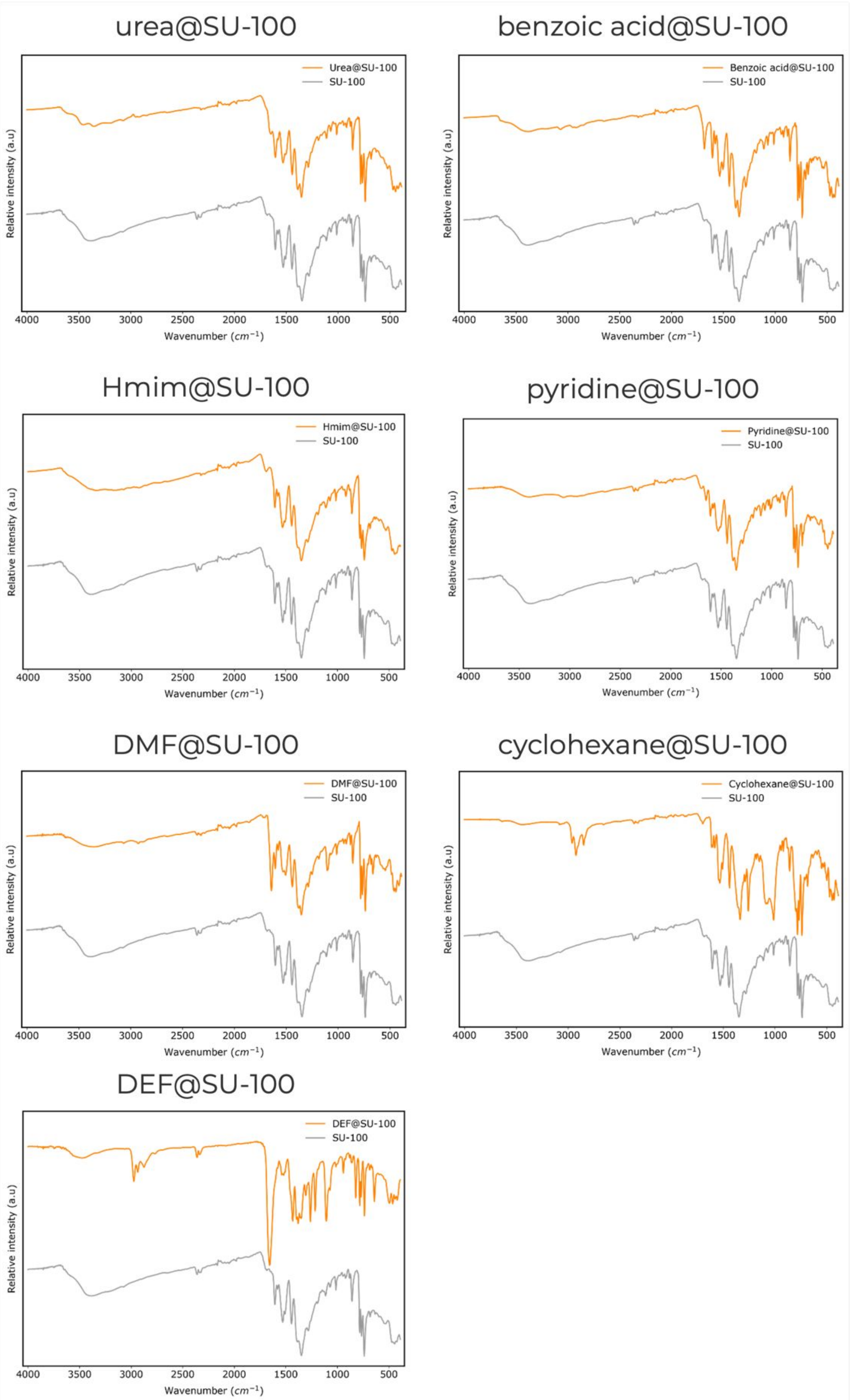

Figure S4. FTIR spectra of SU-100 after guest soaking of pure phase and in solution. No peaks observed at 3000-3700 cm^{-1} for urea@SU-100, benzoic acid@SU-100, Hmim@SU-100, pyridine@SU-100, DMF@SU-100; cyclohexane@SU-100, and DEF@SU-100, indicating a complete exchange of methanol.

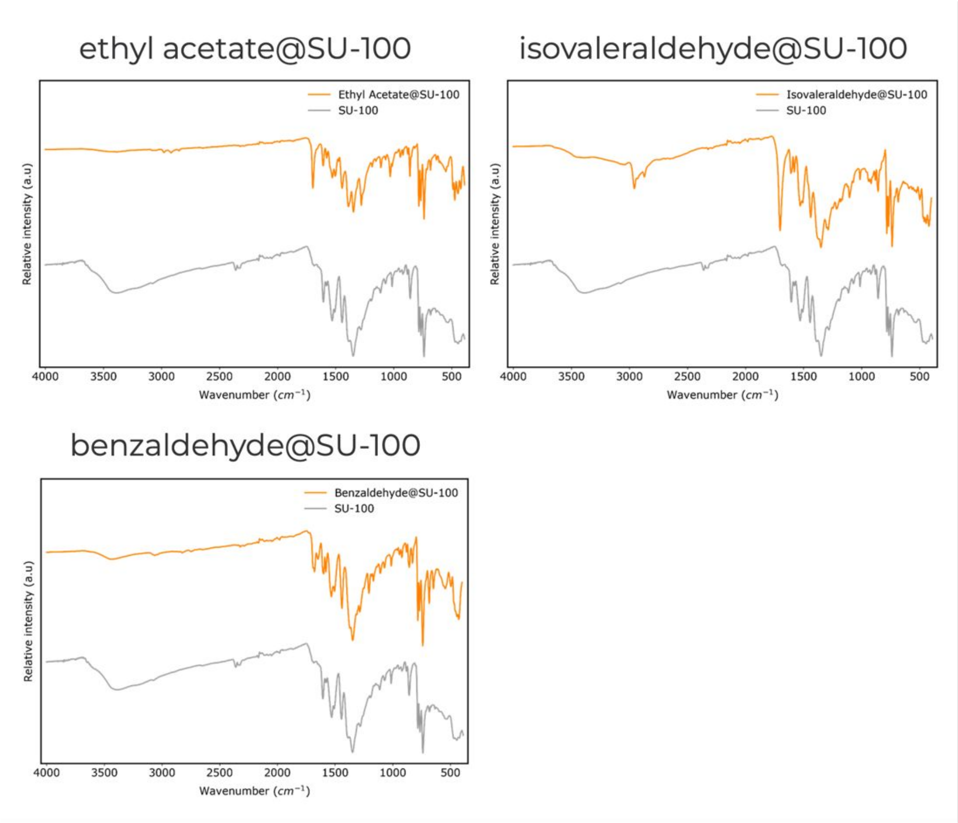

Figure S5. FTIR spectra of SU-100 after guest soaking as vapors. No peaks observed at 3000-3700 cm^{-1} indicating a complete exchange of methanol for ethyl acetate@SU-100, isovaleraldehyde@SU-100, and benzaldehyde@SU-100.

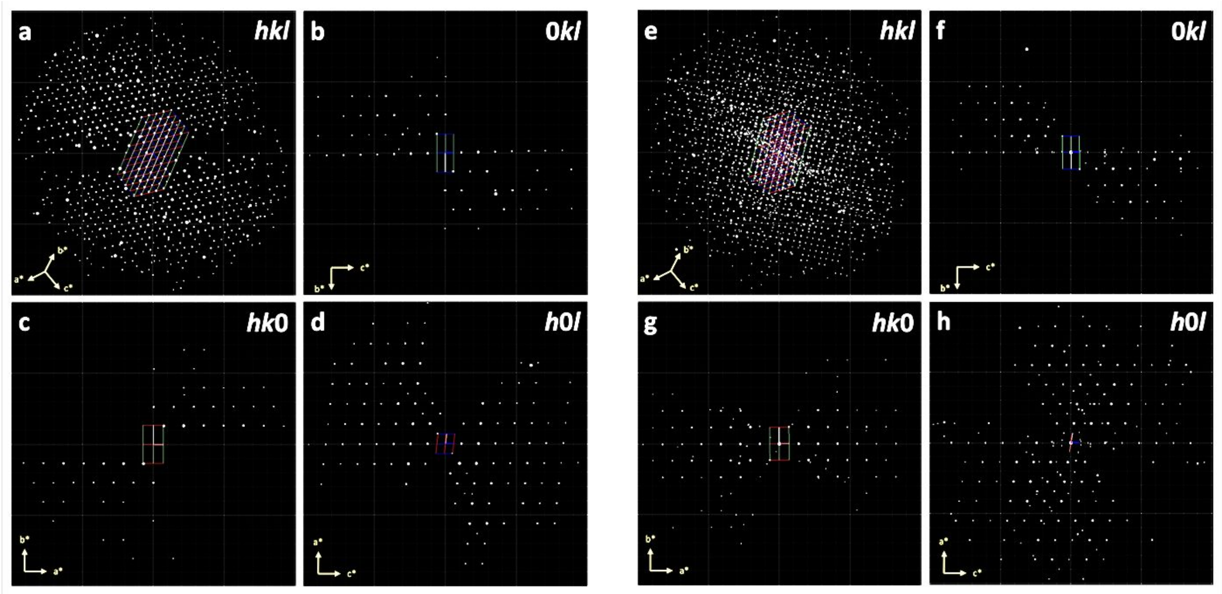

Figure S6. Reciprocal lattice of as-synthesized SU-100 reconstructed from 3D ED data. (a-d) As-synthesized SU-100 showing (a) 3D *hkl* projection and 2D slices (b) 0*kl*; (c) *hk*0; (d) *h*0*l*. (e-h) DEF@SU-100 showing (e) 3D *hkl* projection and 2D slices (f) 0*kl*; (g) *hk*0; (h) *h*0*l*.

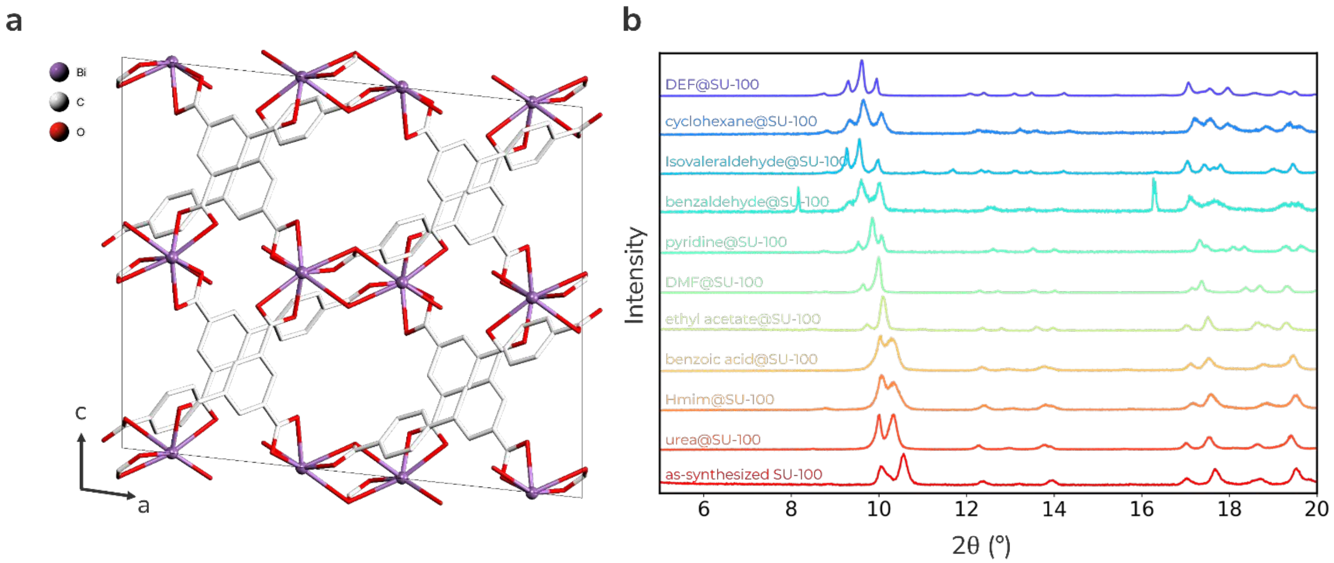

Figure S7. (a) The structure of as-synthesized SU-100 viewed along [010]. Bismuth, carbon, and oxygen atoms are depicted in purple, grey, and red, respectively; (b) PXRD patterns of as-synthesized SU-100 and SU-100 after soaking different guest molecules. The intensities are normalized.

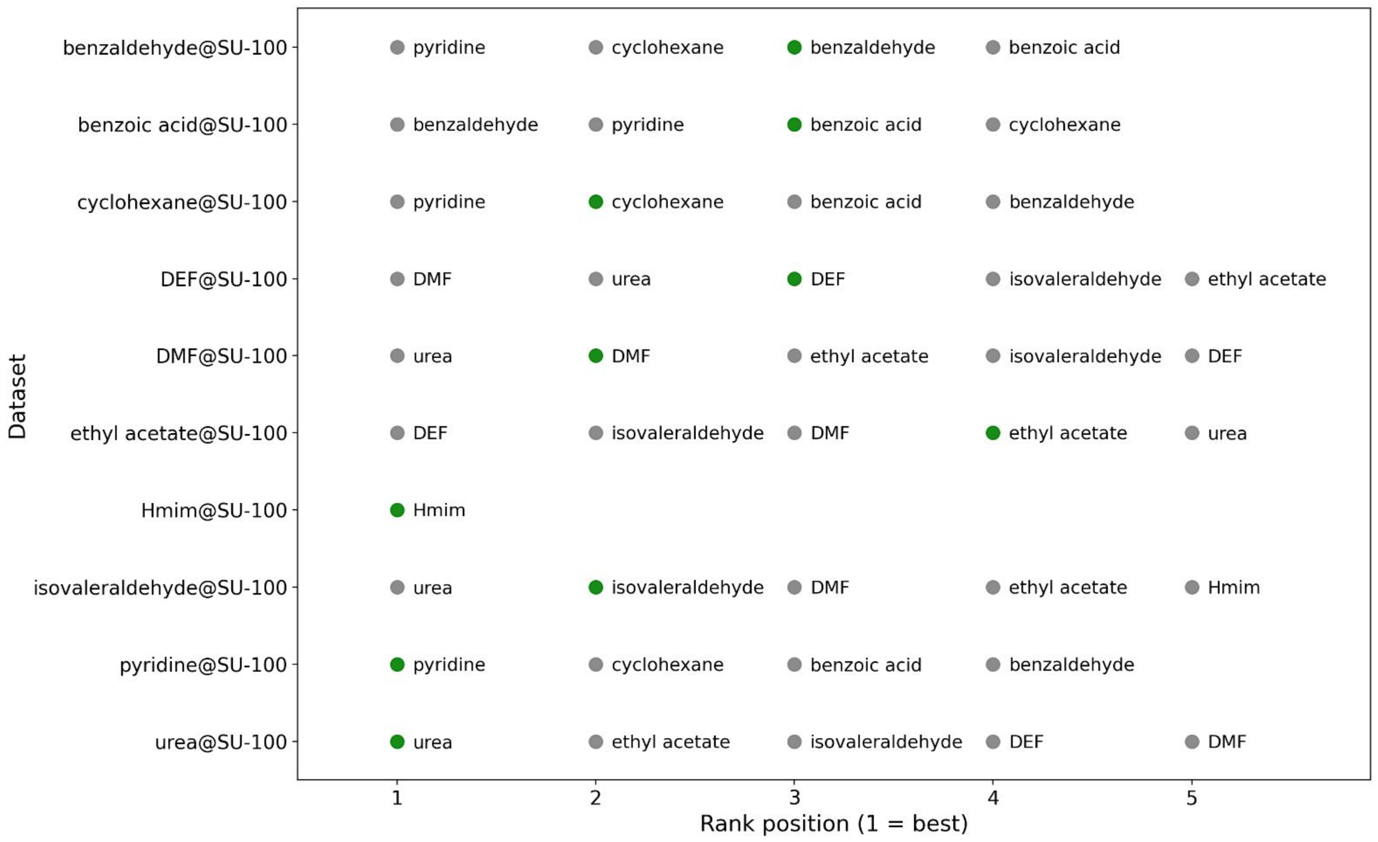

Figure S8. Ranking of the top candidates by AutoSolveX[5]. Max 5 candidates are given for each dataset, and the true molecules are indicated in green.

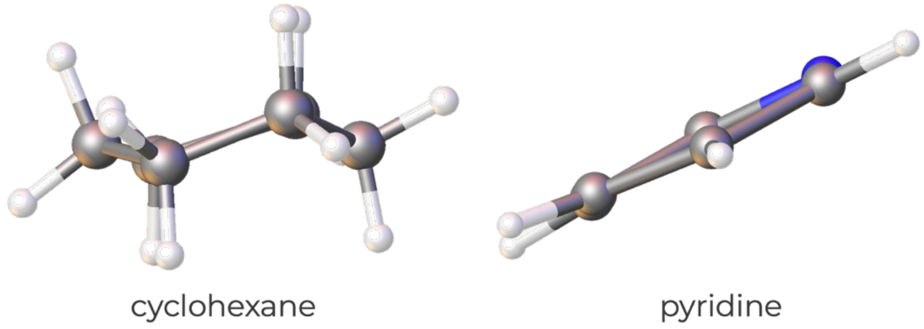

Figure S9. Refined geometries of cyclohexane and pyridine in SU-100.

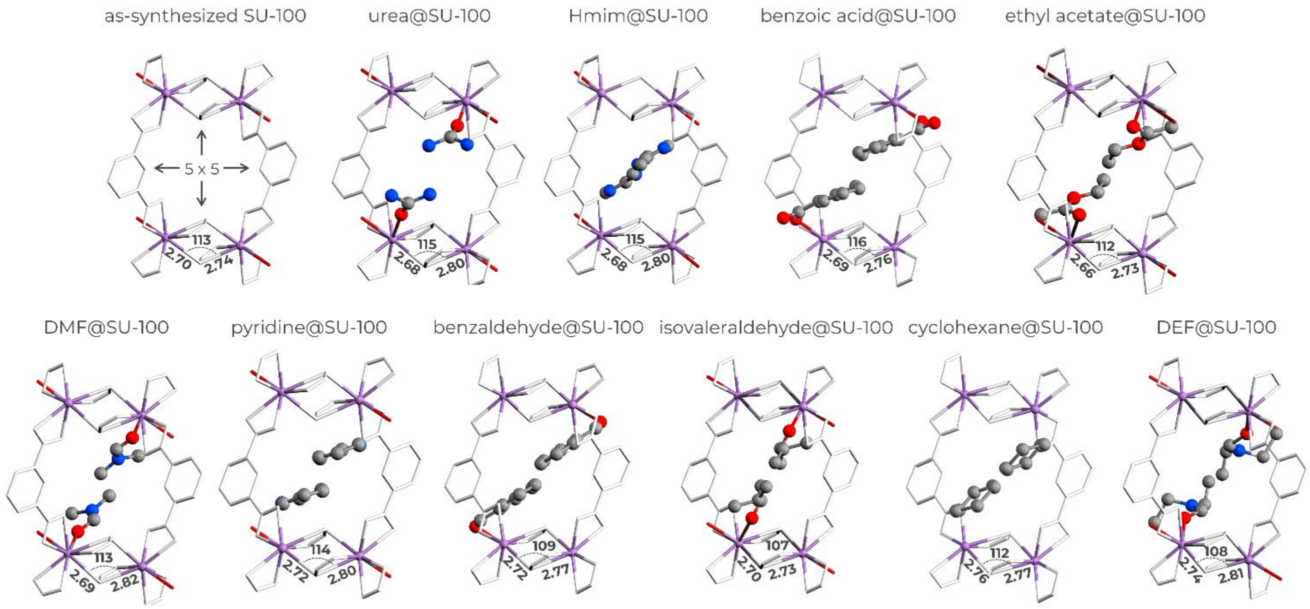

Figure S10. Framework pore structures of as-synthesized SU-100 and SU-100 after soaking of guest molecules, viewed along [010]. Red, blue and grey spheres depict oxygen, nitrogen and carbon atoms in guest molecules. Bismuth is depicted in purple. Oxygen belonging to the water molecule is depicted in red. Marked bond lengths correspond to the Bi−O bond distances (Å), and marked angles correspond to Bi−O−Bi angles (°).

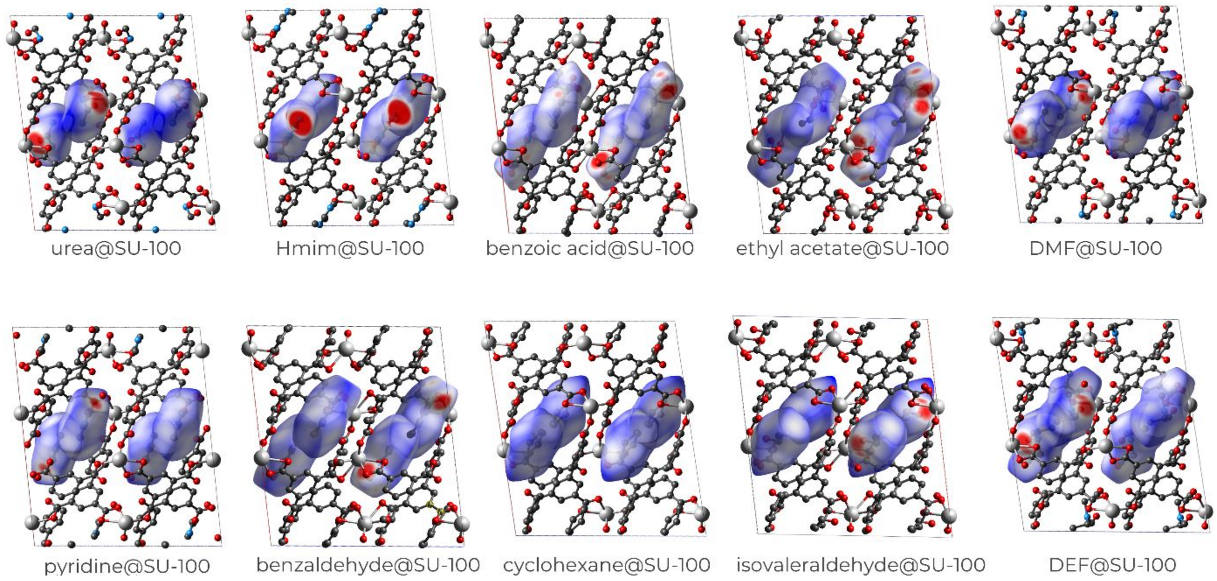

Figure S11. Structures of as-synthesized SU-100 and guest@SU-100, viewed along [010] showing the Hirshfeld surface of the guest molecules and their locations at the intersection of the pores of SU-100. The color scheme used on this surface indicates the contact distance to the framework. Blue surface corresponds to the longer contacts than the sum of the van der Waals radii, white to contacts that are about equal to the sum of the van der Waals radii, and red to the shorter contacts. The scale for the surface was set as dnorm over the range of −0.2889 to 1.3749.[9]

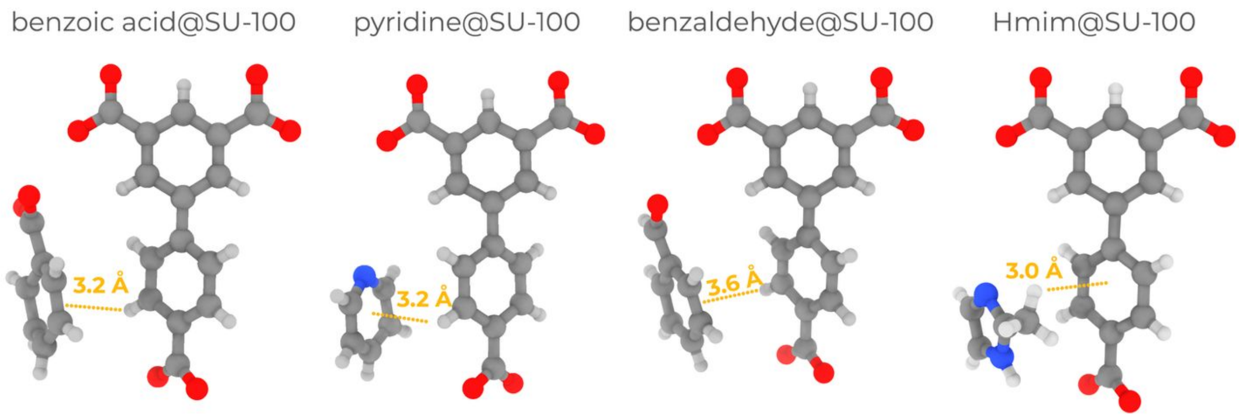

Figure S12. Offset-type π- π stacking interactions between aromatic guests and the SU-100 framework.

References

(1) Grape, E. S.; Xu, H.; Cheung, O.; Calmels, M.; Zhao, J.; Dejoie, C.; Proserpio, D. M.; Zou, X.; Inge, A. K. Breathing Metal-Organic Framework Based on Flexible Inorganic Building Units. *Cryst. Growth Des.* **2020**, *20* (1), 320–329. https://doi.org/10.1021/acs.cgd.9b01266.

(2) Inokuma, Y.; Yoshioka, S.; Ariyoshi, J.; Arai, T.; Hitora, Y.; Takada, K.; Matsunaga, S.; Rissanen, K.; Fujita, M. X-Ray Analysis on the Nanogram to Microgram Scale Using Porous Complexes. *Nature* **2013**, *495*, 461–466. https://doi.org/10.1038/nature11990.

(3) Sato, H.; Yamano, A. Determination of Molecular Structure of Odor Components Based on Crystalline Sponge Method. *Rigaku Journal* **2019**, *35* (1), 2019.

(4) Coelho, A. A. TOPAS and TOPAS-Academic: An Optimization Program Integrating Computer Algebra and Crystallographic Objects Written in C++: An. *J. Appl. Crystallogr.* **2018**, *51* (1), 210–218. https://doi.org/10.1107/S1600576718000183.

(5) Butonova, S.; Chen, Y.; Zou, X. AutoSolveX: A Python-Based Pipeline for Automated Structure Solution, Refinement, and Guest Identification from Diffraction Data. *ChemRxiv* **2026**, No. 0211. https://doi.org/10.26434/chemrxiv.10002125/v1.

(6) Kabsch, W. XDS. *Acta Crystallogr. D Biol. Crystallogr.* **2010**, *66* (2), 125–132. https://doi.org/10.1107/S0907444909047337.

(7) Kabsch, W. Integration, Scaling, Space-Group Assignment and Post-Refinement. *Acta Crystallogr. D Biol. Crystallogr.* **2010**, *66* (2), 133–144. https://doi.org/10.1107/S0907444909047374.

(8) Karplus P. A., Diederichs K. Linking Crystallographic Model and Data Quality. *Science.* **2012**, *336* (6084), 1030–1033. https://doi.org/10.1126/science.1216980.

(9) Spackman, P. R.; Turner, M. J.; McKinnon, J. J.; Wolff, S. K.; Grimwood, D. J.; Jayatilaka, D.; Spackman, M. A. CrystalExplorer: A Program for Hirshfeld Surface Analysis, Visualization and Quantitative Analysis of Molecular Crystals. *J. Appl. Crystallogr.* **2021**, *54*, 1006–1011. https://doi.org/10.1107/S1600576721002910.
